# Understanding healthcare utilisation for aboriginal people in New South Wales prisons with histories of self-harm and suicidal behaviour: a retrospective cohort study

**DOI:** 10.1186/s12889-025-25917-w

**Published:** 2025-12-16

**Authors:** Reem Zeki, Sharlene Kaye, Grantley Creighton, Mark V.A. Howard, Robyn Shields, Andrew Ellis, Gary Nicholls, Wendy Hoey, Vindi Nanayakkara, Julia Bowman

**Affiliations:** 1 Research and Evaluation Team, Justice Health and Forensic Mental Health Network, 1300, NSW 2036 Malabar, Australia; 2 School of Medicine and Public Health, University of Newcastle, Newcastle, Australia; 3 National Drug and Alcohol Research Centre, University of New South Wales, Sydney, Australia; 4 Aboriginal Health, Justice Health and Forensic Mental Health Network, Sydney, Australia; 5 Corrections Research Evaluation and Statistics, Corrective Services NSW, Sydney, Australia; 6 Forensic Mental Health, Justice Health and Forensic Mental Health Network, Sydney, Australia; 7 Executive Medical Services, Justice Health and Forensic Mental Health Network, Sydney, Australia; 8 Justice Health and Forensic Mental Health Network, Sydney, Australia

**Keywords:** Self-harm, Suicide, Aboriginal people, Prisons, Service utilisation, Australia, Real-world data

## Abstract

**Background:**

People in prison are at increased risk of suicide. Aboriginal people are overrepresented in Australian prisons, and their self-harm/suicide risk may be complicated by experiences of trauma, colonisation, loss of land and culture, and social injustices. This study aims to investigate mental health morbidities and in-prison service utilisation of Aboriginal people with histories of self-harm and/or suicidality.

**Methods:**

Historical cohort study utilising Justice Health and Forensic Mental Health Network routinely collected data, including records of Aboriginal people entering NSW public prisons from 2015 to mid-2024. Records included Reception Screening Assessments (RSA), Patient Administration System appointments and alerts, and patient transfers to external hospitals.

Descriptive statistics were produced for people’s characteristics, appointments, type of professional/clinician seen, alerts, and hospital transfers. Multivariable logistic regression was used to investigate the association between self-harm and/or suicidal behaviour (SHSB) reported at reception and mental health appointments.

**Results:**

The study includes 42,161 RSAs for 15,583 Aboriginal people. A history of SHSB was reported in 10,253 RSAs. Of the study population, 2152 people reported having ever attempted self-harm/suicide at one reception without disclosing this information in a later reception. Depression and anxiety were the most prevalent mental health conditions reported by people with a history of SHSB.

Of all appointments booked within four weeks of reception for people reporting SHSB, only 447 appointments (0.3%) were with an Aboriginal Health Worker. People who reported SHSB were 37% more likely to have a mental health appointment booked within four weeks of reception compared to people who did not report SHSB. Of mental health appointments booked within four weeks, 51.2% involved individuals under Risk Intervention Team management, with 59.0% following RSAs where SHSB was reported. There were 452 hospital transfers due to self-harm or suspected suicide attempts, with 20.1% occurring within four weeks of reception.

**Conclusion:**

Self-reporting self-harm/suicide at reception, on its own, is an unreliable predictor of future risk. Better identification of people with SHSB histories could support delivery of safer care for Aboriginal people. Establishing a therapeutic environment and offering comprehensive, culturally sensitive healthcare can help lower the risk of self-harm and suicide among people in prison.

## Introduction

Suicide is a global public health problem, with around 720,000 deaths occurring annually due to suicide [1]. In Australia, the age-standardised rate of death due to suicide in 2023 was 11.8 per 100,000 population, representing 1.8% of all causes of death [2]. In New South Wales (NSW), the age standardised rate of suicide deaths in 2023 was 9.9 per 100,00; and the rate of Aboriginal deaths by suicide in 2023 was 28.2 per 100,000 [3, 4]. Although suicide affects all population groups, suicide risk is highly varying and disproportionately affects the most marginalised and disadvantaged populations [5, 6]. People in contact with the justice system are at higher risk of death by suicide than the general population [6, 7]. People in prison are 2.2 times more likely to die by a self-inflicted injury compared to the general population [8]. In Australia, the proportion of deaths in custody due to suicide over a 5-year period decreased from 38.9% between 1992 and 1997 to 10.4% between 2017 and 2022 [2]. Despite this decrease, the proportion of deaths in custody due to suicide is still more than five times that of the Australian general population (10.4% vs. 1.8%).

Suicide in prison is associated with several risk factors. Suicidal ideation is the strongest risk factor associated with suicide in prison, with around 50% of people with suicidal ideation subsequently attempting suicide [5, 9–12]. Additionally, people with recent suicidal ideation are 16 times more likely to attempt suicide in prison [13]. Other risk factors include having a history of suicide attempts and non-suicidal self-harm, with each contributing to a six-fold increase in the likelihood of suicide attempts (odds ratio [OR] = 5.95, 95% confidence interval [CI] 3.17–11.16) and (OR = 6.16, 95% CI 4.98–7.62), respectively [13]. Among adults in NSW prisons, one third reported having ever thought about committing suicide and 17.8% reported actual suicide attempts [14]. Additionally, one in five (21%) people entering prison reported self-harm behaviour at some stage in their life [15]. Previous research has explained the increased risk of suicide behaviour and self-harm among people in prison by two main factors:

1. Factors related to people in contact with the justice system: People in prison are affected by social and health inequalities. Factors such as poor and unstable housing, lower education levels and lack of employment are associated with an increased risk of suicidal behaviour and drive contact with the justice system [5, 16]. A history of childhood sexual, physical, and emotional abuse, which is more prevalent among people in prison, is also associated with an increased risk of suicide in prison, with people who experience child abuse being at three-fold increased risk of committing suicide in prison [5, 13, 16]. Additionally, people in prison are at higher risk of psychiatric disorders, which are strongly associated with self-harm and suicidal behaviour in prison [13, 16]. People who receive psychiatric treatment in prison or those who had such treatment prior to prison entry are eight and five times more likely, respectively, to commit suicide in prison [13]. Substance use disorder is another factor which is highly prevalent among people in contact with the justice system and associated with suicide risk [5, 17].
2. Factors related to the prison environment and stressors: Factors such as solitary confinement, physical and sexual victimisation, and lack of social support are associated with an increased risk of self-harm and suicide attempts [5, 13, 16]. In addition to the prison environment, certain stressors and periods are associated with an increased risk of self-harm and suicide, specifically the early weeks after prison reception and the remand period, when people feel separated from their family and friends, go through repeated court visits, at sentencing time, and are uncertain about their future [5, 18].

Aboriginal and/or Torres Strait Islander people (hereafter, respectfully referred to as Aboriginal people) are overrepresented in the prison population, representing 36% of the Australian prison population compared to only 3.8% of the Australian general population (19, 20). An Aboriginal investigator led study (Dudgeon et al, 2017) discussed the context and the causes of suicide among Aboriginal people concluded that in addition to the risk factors associated with suicide in the general prison population, Aboriginal people are at risk of suicide due to a complex web of factors including the biological, personal and social effects of trauma (21), intergenerational impacts of colonisation, loss of land and culture, discrimination, social exclusion and disadvantage, and denial of social justice (21, 23). Arguably, colonial models of health care likely compound risk. 

In Australia, between 1 July 2022 and 30 June 2023 there were 21 Aboriginal deaths in prison custody representing the highest number of deaths in prison custody since 1979-1980 (24). The number of Aboriginal deaths in 2022-2023 account for 30% of all deaths in custody over this period, which is higher than the average 18% recorded since 1979-1980 (24). Of the 21 deaths in prison custody that occurred in 2022-2023, cause of death was recorded and able to be ascertained in 13 cases, 5 (38.5%) of which were self-inflicted deaths (24). In NSW, based on the report by the NSW State Coroner into first Nations People’s Deaths in Custody in NSW: 2008-2018, there were 34 Aboriginal deaths in custody in NSW (25). Intentional self-harm accounted for 29% (n=10) of these deaths, with the majority occurring while people were on remand (25).

In NSW, the prison system comprises 33 publicly operated and 3 privately operated correctional centres. This studyincludes data from all public prisons, which are managed by Corrective Services NSW (CSNSW). Currently,approximately 6500 staff are employed in CSNSW correctional centres, of whom 3.2% identify as Aboriginal or TorresStrait Islander [26].

According to the NSW Crimes (Administration of Sentences) Regulation 2014, people in public prisons are entitled to atleast two hours of outdoor exercise per day, except for those confined to their cells, who must be allowed at least onehour of exercise daily [27]. People in NSW prisons spend an average of 8.3 h out of cells each day [28].

People held on remand are permitted two visits per week, while visiting arrangements for sentenced people vary byfacility and are determined by the Governor of each correctional centre [29]. In addition to in-person visits, people inNSW prisons have routine access to phone calls with friends and family, including increasingly frequent use of thisfacility via in-cell digital tablets since their introduction in recent years [30].

CSNSW offers a range of programs tailored to the needs of people in custody. These include programs specificallydesigned for people on remand and those serving short sentences, as well as general programs accessible to all people inprison. In addition, targeted interventions are available, such as: drug and Alcohol programs, aggression/violenceprograms, countering Violent Extremism programs, sex offender programs, young adult offender programs and safedriving programs [31].

The management of people in NSW prisons identified as at risk of self-harm or suicide, upon reception to prison orduring their time in custody, includes the mandatory notification of such risk to all relevant staff and development of arisk management plan appropriate to the level of risk. In response to a notification of self-harm or suicide risk, the RiskIntervention Team (RIT) convenes to formulate a management plan, which includes the appropriate cell-placement andobservation schedule. The RIT is responsible for the ongoing assessment of risk and review of management plans, andreferral to specialist assessment or treatment services, where appropriate.

The Justice Health and Forensic Mental Health Network (Justice Health NSW) provides health care for people (adultsand young people) in public prisons [32]. It is a state-wide specialty health network that provides multidisciplinaryhealth services to more than 30,000 people annually in several settings, including correctional centres, youth justicecentres, police cells, courts, inpatient and community settings [32]. Understanding how Aboriginal people with a historyof self-harm or suicidal behaviour use services is essential for achieving zero suicides and reducing the disparities inhealth outcomes between Aboriginal and non-Aboriginal people in custody, in accordance with the National Agreementon Closing the Gap. These are key objectives of the Justice Health NSW 10-year Strategic Plan 2023-32 [32]. Byidentifying gaps in service provision and usage, we can develop a more supportive and effective system.

Previous studies investigating self-harm and suicide among Aboriginal people in prison have focused on self-harm andsuicide rates, risks and protective factors [22, 33, 37]. To the authors’ knowledge, this is the first real-world study thatinvestigates the service utilisation of Aboriginal people in prison with histories of self-harm and/or suicidal behaviours (SHSB).

This study aims to investigate mental health morbidities and in-prison service utilisation of Aboriginal people withhistories of self-harm and/or suicidality. We aim to address the gap in the literature on the in-prison health care providedfor this at-risk population. We believe the findings of this study will provide evidence-based insights to support theplanning and delivery of culturally informed healthcare for populations with complex and unmet health needs.

## Method

This paper was led by two Aboriginal and eight non-Aboriginal co-authors, whose expertise in Aboriginal health, mental health, and the health of people in prison shaped its development. Additionally, the results of this paper were presented and discussed with the People in NSW Public Prisons: Heath Status and Service Utilisation project Aboriginal Community Reference Group. This work has been approved for publication by the Aboriginal Health and Medical Research Council Human Research Ethics Committee.

### Study population and duration

Records of Aboriginal people who entered NSW public prisons from 1 January 2015 to 30 June 2024. Multiple records for each person were included in the study. Self-report of Aboriginal identity at reception was used to identify records of Aboriginal people. As multiple receptions per person were used in this study, if a person is self-identified as Aboriginal in any reception all their other reception records were included in the analysis.

### Data source

Three real-world routinely collected administrative data sets were used for the analysis of this study. These data sets are part of Justice Health NSW electronic data systems.

1. *Reception Screening Assessment (RSA)* is part of Justice Health electronic Health System (JHeHS). It is a health assessment conducted for all adults upon entry into custody (i.e., within 24 h of reception). If the RSA is unable to be completed within 24 h of a person entering custody, a reason must be provided. The RSA form is a structured screening tool completed by a registered or enrolled nurse. The aim of the RSA is to identify immediate health related needs for people entering custody with the focus on physical and mental health including self-harm and suicide risk assessment, alcohol and drug use, women’s health, as well as population and preventative health risks.
2. *Patient Administration System (PAS)* is an electronic system where patients’ interactions are recorded and managed. It is used by Justice Health NSW to manage outpatient appointments and inpatient information such as admissions, transfers, and discharges. PAS appointment data up to 31 July 2024 was included in this data set to allow for a follow-up period of at least four weeks. Medical alerts are flags created in PAS. Alerts can be clinical, created for certain conditions, allergy, medication or risks, non-clinical alerts can be administrative alerts or initiated for patient involved in certain programs. If an alert has an end date it considered in-active alert. PAS alert data up to November 2024 was included in the analysis for the study.
3. *Daily Update – Patient Transfer to an External Hospital data* is part of JHeHS and is available from October 2020. It includes all unplanned patient transfers to an external hospital. Planned outpatient appointments are not included in these data.

### Study group and comparison group

The study group includes RSA episodes where people self-report a history of SHSB. As part of the RSA suicide risk assessment section, people entering custody were asked (1) Have you ever tried to hurt yourself? and (2) Have you ever tried to end your life? An RSA was included in the study group if “yes” was the answer to one or both questions (SHSB = yes). An RSA was included in the comparison group if “no” was the answer for both questions (SHSB = no). If information on both questions was missing, or one of the questions had missing data and the other was “no”, then self-harm/suicidal behaviour was considered to be “not stated”.

#### Self-harm/suicide attempts within one month prior to prison reception

People who reported previous self-harm and/or suicide attempts were asked about the time of the last attempt. As part of the change in the RSA form on 1 February 2021, this question was changed from a free text response format to a categorical format. Prior to 1 Feb 2021, the authors flagged any episode where people indicated their last attempt occurred within one month prior to their prison reception. Phrases such as “a few days ago”, “a couple of weeks ago”, “last month”, and “1 month ago” were considered as self-harm and/or suicide attempts within one month prior to prison reception. From 1 February 2021, RSA episodes where people who reported a self-harm and/or suicide attempt indicated that their last attempt was “in the past week” or “1–4 weeks ago” were flagged as self-harm/suicide attempts within one month prior to prison reception.

### Measurements and calculations

#### Sociodemographic characteristics

Presented for people who reported SHSB at least once and those who did not report SHSB in any of their RSAs. Age was based on the first RSA completed in a public prison within the study duration.

#### Mental health comorbidities

Based on self-reported data at reception. People can report more than one mental health condition in one RSA. The mental health section of the RSA form was changed in February 2021. As a result, mental health morbidities were produced for two separate periods: prior to 1 February 2021, and from 1 February 2021 onwards.

#### Mental health appointments within four weeks after reception

Includes booked mental health appointments where the appointment date is at, or within 28 days of, the reception date.

#### Type of professional carer

Produced for all booked appointments (i.e., all appointment types) within four weeks of reception.

#### Self-harm/suicide alerts

Any patient who is identified as at risk of self-harm is flagged using an “alert” placed in the PAS. These alerts are visible in both PAS and JHeHS. There are five self-harm and suicide alerts. Self-harm alerts include self-harm risk, threats, or deliberate self-harm. Suicide alerts include a history of suicide attempts and current suicide attempts. Alert were included in the analysis when (1) the alert’s start date is at or after the assessment date of an RSA episode and before the assessment date of the next RSA episode (new alert) or (2) the alert’s start date is within a previous RSA episode and missing an alert end date (active old alert).

#### Risk integration team (RIT) alert

A Mandatory Notification (RIT) alert with start date at or after the assessment date of an RSA episode and before the next RSA episode (new alert).

#### RIT appointments

Are managed by the RIT which consists of two CSNSW members and one Justice Health NSW staff. Three types of RIT appointments are recorded in PAS data: (1) RIT new, where patient placed under RIT management; (2) RIT review, where the risk is reviewed by the RIT; and (3) RIT termination, where patient is no longer under RIT management.

#### Transfer to an external hospital

Any unplanned transfer to an external hospital where the external hospital transfer date is at or after the assessment date of an RSA episode and before the assessment date of the next RSA episode. Transfer to an external hospital for a self-harm or suspected suicide attempt was determined using four variables: (1) incident type; (2) provisional diagnosis; (3) other provisional diagnosis; (4) discharge diagnosis. If self-harm or a suspected suicide attempt were recorded in any of the four variables the cause of the transfer was flagged as a transfer for a self-harm or suspected suicide attempt.

#### Hospital stay

Calculated for hospital admissions only. Hospital admission date and discharge date were used to calculate number of days in hospital. If hospital admission date was missing, hospital assessment date was used as a proxy for hospital admission date.

### Statistical analysis

Descriptive statistics (number and proportions) were produced for people’s characteristics, mental health appointments, type of professional carer, alerts, and transfer to an external hospital. Chi Square test was used to compare the proportions of sex and age groups, unplanned hospital transfer and unplanned hospital transfer within four weeks between those who ever reported SHSB and those who didn’t. Mann- Whitney U test was used to compare the median time from reception to the RIT appointments. Multivariable logistic regression was used to investigate the association between self-harm/suicidal behaviour reported at reception and booked mental health appointments. Odds Ratios and 95% CI were produced.

Variables with *p* < 0.20 were included in the initial logistic regression model [38]. Following backward elimination, the final model retained only those variables that remained statistically significant (*p* < 0.05).

The final logistic regression model includes the following covariates: age groups, sex, referral to RIT at RSA, reported mental health conditions - depression, anxiety, schizophrenia, bipolar disorder, drug-induced psychosis, post-traumatic stress disorder, oppositional defiant disorder, and attention deficit hyperactivity disorder.

### Ethics

This study is part of the People in NSW Public Prisons: Heath Status and Service Utilisation project. This project is undertaken in accordance with the National Statement on Ethical Conduct in Human Research 2023, which outlines ethical principles for studies involving human participants in Australia and is underpinned by the Declaration of Helsinki. Ethics approval for the project was granted from Justice Health and Forensic Mental Health Network Human Research Ethics Committee (Reference number: 2020/ETH01927) and the Aboriginal Health and Medical Research Council Human Research Ethics Committee (Reference number: 1719/20).

## Results

**Fig. 1:**
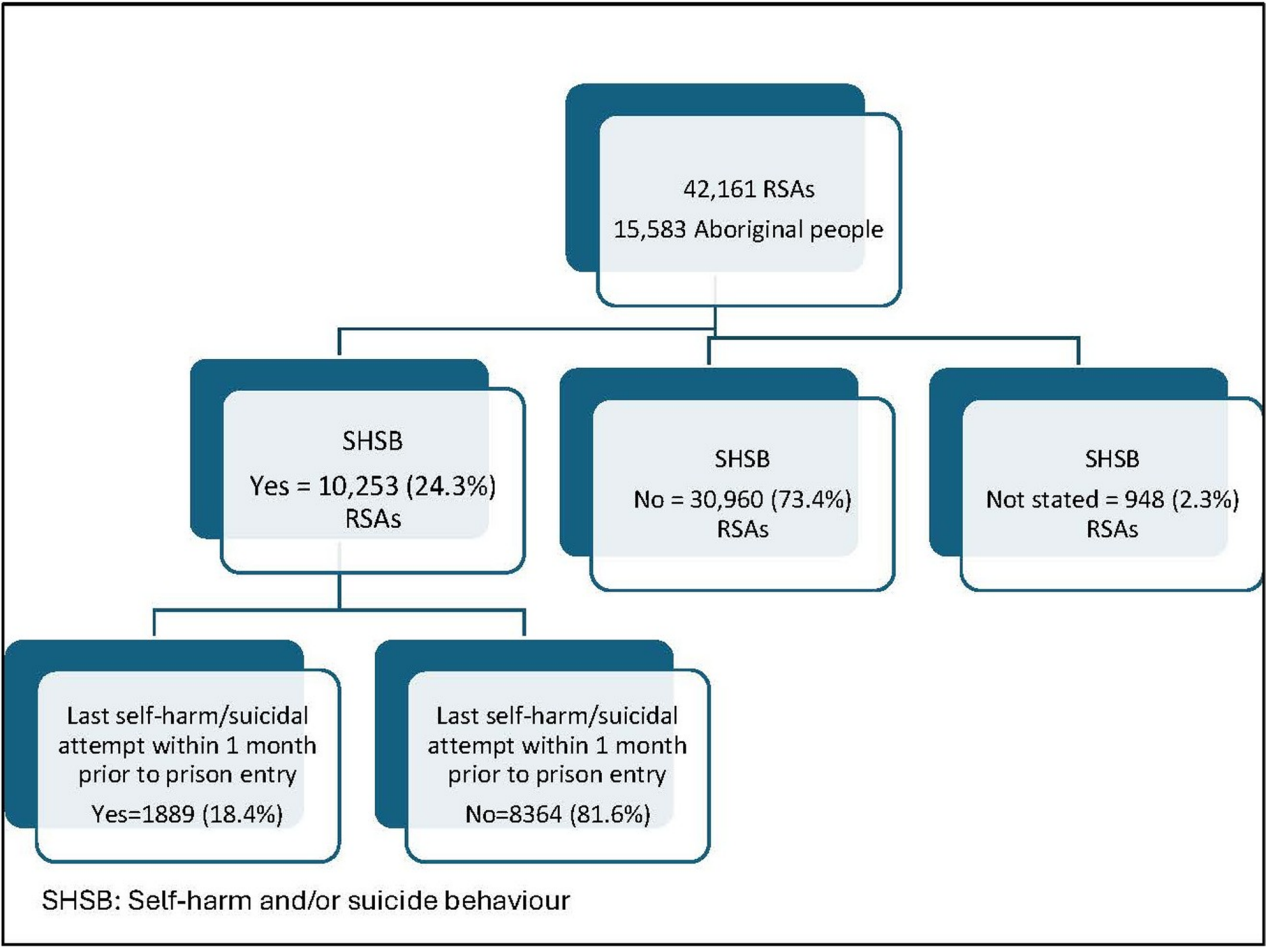
Self-harm/suicidal behaviour among the study population

Between January 2015 and June 2024, there were 42,161 receptions (RSAs) for 15,583 Aboriginal people, with 58.6% of people entering custody more than once during this period. More than one third (37.7%, *n* = 5868) of Aboriginal people entering custody reported SHSB on at least one reception occasion (22.1% reported self-harm and 26.9% reported suicide attempts), with such behaviours reported in almost a quarter (24.3%, *n* = 10,253) of the RSAs (Fig. 1). Of those, 2152 people reported having ever attempted self-harm and/or suicide at one reception and did not disclose this information in a later reception into custody. Out of 10,253 RSAs where people reported SHSB, 1889 (18.4%) attempts were made within the month prior to prison reception.

### Sociodemographic characteristics and mental health (co)morbidities

Table 1 shows the sociodemographic characteristics of the study population, based on the first reception episode during the study period (23.5%) of people who reported SHSB at least once during the study period were women. This proportion is higher than the proportion of women among all prison entrants across the study period (19.2%). More than one third (36.0%) of those who reported SHSB were younger than 25 years, a higher proportion than that of young people entering prisons across the study period (29.8%) (Table 1). The proportion of young people was similar among men and women who reported SHSB at least once (36.5% and 34.5%, respectively).

**Table 1:** Sociodemographic characteristics of study population

	Reported SHSB at least once								
	Yes		No		Not stated		All		*P* value^a^
	*N*	%	*N*	%	*N*	%	*N*	%	
Sex									
Female	1376	23.5	1588	16.5	20	23.8	2984	19.2	*P* < 0.001
Male	4492	76.6	8040	83.5	64	76.2	12,595	80.8	
Not stated	0	0.0	3	0.0	0	0.0	3	0.0	
Age groups (years) at first reception									
Less than 20	690	11.8	767	8.0	5	6.0	1463	9.4	*P* < 0.001
20 to 24	1424	24.3	1739	18.1	11	13.1	3173	20.4	
25 to 29	1161	19.8	1720	17.9	13	15.5	2894	18.6	
30 to 34	927	15.8	1676	17.4	14	16.7	2615	16.8	
35 to 39	701	12.0	1365	14.2	18	21.4	2085	13.4	
40 to 44	470	8.0	981	10.2	6	7.1	1456	9.3	
45 to 49	296	5.0	727	7.6	9	10.7	1033	6.6	
50 to 54	136	2.3	375	3.9	4	4.8	515	3.3	
55 and over	63	1.1	280	2.9	4	4.8	348	2.2	
Not stated	0	0.0	1	0.0	0	0.0	1	0.0	
Total	5868	100	9631	100.0	84	100	15,583	100.0	

a) Excluding not stated values

SHSB: Self-harm and/or suicidal behaviour

Age based on first reception (first RSA) in public prisons within the scope of the study period

Tables 2a and 2b explore the mental health morbidities reported at reception during the study period by Aboriginal people with a history of SHSB.

For the period from 1 January 2015 to 31 January 2021, ever having treatment for a mental health condition was reported in 78.1% of the RSA episodes, with depression being the most commonly reported mental health condition to have been treated (Table 2a).

For RSA episodes between 1 February 2021 and 30 June 2024 where people reported SHSB, the most frequently reported mental health condition was depression (76.2%), followed by anxiety (69.2%) and schizophrenia (32.3%). Of those RSAs 2854 (79.1%) where people reported more than one mental health condition. Of RSA episodes where people reported SHSB, more than half of people undergoing assessment reported receiving medication for their mental health condition and 30% had been admitted to hospital for a mental health problem. No mental health condition was reported in only 7.3% of episodes where people reported SHSB (Table 2b).

**Table 2:** A: mental health comorbidities (1 Jan 2015-31 Jan 2021). B: Mental health comorbidities (1 Feb 2021-30 Jun 2024)

A: Mental health comorbidities (1 Jan 2015-31 Jan 2021)		
RSAs where people reported SHSB (total = 6643)	*N*	%
RSA with reported treatment for a mental health condition		
Yes	5187	78.1
Mental health condition treated		
*Depression*	*2871*	*55.4*
*Anxiety*	*1458*	*28.1*
*Schizophrenia*	*1209*	*32.3*
*Bipolar disorder*	*856*	*16.5*
*Post-traumatic stress disorder*	*516*	*10.0*
No	1345	20.3
Not stated	111	1.7
Total	6643	100.0

B: Mental health comorbidities (1 Feb 2021-30 Jun 2024)		
RSAs where people reported SHSB (total =3610) RSAs with reported mental health condition	*N*	%
Depression	2750	76.2
* Had depression symptoms in the month before prison entry*	*2136*	*77.7*
Anxiety	2499	69.2
* Had anxiety symptoms in the month before prison entry*	*2025*	*81.0*
Schizophrenia	1171	32.4
* Had schizophrenia symptoms in the month before prison entry*	*828*	*70.1*
Bipolar disorder	824	22.8
* Had bipolar disorder symptoms in the month before prison entry*	*606*	*73.5*
Substance Induced Psychosis	629	17.4
* Had drug * *Substance Induced Psychosis* * symptoms in the month before prison entry*	*223*	*35.5*
Other	1483	41.1
* Had other mental health condition symptoms in the month before prison entry*	*1006*	*67.8*
No mental health condition	264	7.3
RSAs with reported treatment for a mental health problem		
Medication	2132	59.1
Therapy or Counselling	813	22.5
Seen by a psychiatrist	1278	35.4
Seen by community mental health team	518	14.4
Been under a Community Treatment Order	183	5.1
Admitted to hospital for a mental health problem	1098	30.4

SHSB: Self-harm and/or suicidal behaviour

For RSAs where people reported SHSB More than one mental health condition can be reported in one RSA More than one mental health treatment can be reported in one RSA

As part of the reception screening assessment and if indicated based on the results of their mental health and self-harm/suicide risk assessment, people may be placed under RIT management (commonly known in the custodial setting and hereafter referred to as (on RIT)), referred to a mental health nurse, or both. Of RSAs where people reported SHSB, 30.03% were placed on RIT. Where data on referrals to a mental health nurse is available (from 2021 onwards), records show that 88.5% were referred to a mental health nurse and 35.4% were placed on RIT and referred to a mental health nurse. These proportions were 4.1%, 56.1% and 4.4%, respectively, for RSAs where people did not report SHSB.

### Mental health appointments within four weeks after reception

Of the reception episodes included in this study, 40.9% (*n* = 17,242) were episodes where people had at least one mental health appointment booked within four weeks of reception. Of episodes where people reported SHSB, more than half (63.9%) had at least one mental health appointment within four weeks of reception. In comparison, 32.4% of reception episodes where people did not report SHSB were followed by a mental health appointment booking within four weeks.

In total there were 60,834 mental health appointments booked within four weeks after reception, representing 10.5% of all booked medical appointments in PAS within four weeks for Aboriginal people who entered NSW public prisons during the study period (16.7%) of all appointments for Aboriginal people with a SHSB history were mental health appointments: for people who did not report SHSB, 7.3% of all appointments were for mental health appointments.

Table 3 presents the results of the multivariable logistic regression. It shows that people who reported SHSB were 37% more likely to have mental health appointments booked within four weeks of the reception date compared to people who did not report SHSB (AOR 1.37, 95% CI 1.34–1.40) (Table 3). Sub-analysis to compare booked mental health appointments within four weeks of reception for people who reported self-harm and/or suicide attempts within four weeks prior to prison entry compared to those who report SHSB but with no attempts within four weeks prior to prison entry did not show a significant difference (AOR 1.01, 95% CI 0.98–1.04).

**Table 3:** Multivariable logistic regression analysis of booked mental health appointments within 4 weeks

Reported SHSB at reception	Mental health appointments		Univariable analysis		Multivariable analysis	
	*N*	%	Unadjusted OR	95% CI	AOR^a^	95% CI
Yes	27,610	16.71	2.54	2.50–2.59	1.37	1.34–1.40
No	28,914	7.32	Reference group			

a) Adjusted for sex, age groups, reported mental health conditions (depression, anxiety, schizophrenia, bipolar disorder, drug-induced psychosis, post-traumatic stress disorder, oppositional defiant disorder, and attention deficit hyperactivity disorder),Mandatory Notification Form (MNF)-RIT at reception

*AOR* Adjusted odds ratio, *SHSB* Self-harm and/or suicidal behaviour

More than half (51.2%) of mental health appointments booked within four weeks for people who reported self-harm and/or suicidality at reception were RIT appointments. These appointments include new RIT appointments, RIT reviews, and RIT termination (meaning patients are discharged from this close monitoring process). Mental health reviews and mental health new assessments represented 26.0% and 9.2% of all booked mental health appointments within four weeks, respectively.

The majority (86.2%) of mental health appointments were attended, 7.3% were not attended, and only 4.2% were cancelled. Of the 1172 cancelled appointments, 19.7% were cancelled because the patient was released from custody and 16.0% because the patient was transferred to another correctional centre at the time of the appointment. Thirty-nine appointments (3.3%) were cancelled by a health professional and 38 (3.2%) were cancelled by CSNSW.

Data for delivery mode is available from 2018: face-to-face appointments represented 80.1% of the attended mental health appointments and 6.4% were audio or audiovisual appointments. The majority (91.7%) of the mental health intake appointments were face-to-face. For mental health new assessment appointments, 69.9% were face-to-face and 15.0% were audiovisual. Among mental health appointments completed by primary health professional, 74.2% were face-to-face appointments, 6.7% were audiovisual, and 18.5% were without a client.

Of all appointments booked for people who reported SHSB within four weeks of reception, mental health-related or otherwise, the majority (92.2%) were with nurses (Table 4). A small number of appointments were with medical professionals (7314, 4.4%), 2440 (1.5%) appointments were with psychiatrists and psychologists, and only 447 appointments (0.3%) were with an Aboriginal Health Worker.

**Table 4:** Type of professional carer seen for appointments within 4 weeks of reception

Professional carer type	Number	Percent
Aboriginal Community Health Worker	426	0.3
Aboriginal Health Practitioner	21	0.0
Addiction Medicine Specialist	1220	0.7
Community Worker	867	0.5
Psychiatrist	2365	1.4
Psychologist^a^	75	0.0
Nurse^b^	152,368	92.2
Other Allied Health Specialist^c^	678	0.4
General Practitioner	4638	2.8
Healthcare Practitioner	6	0.0
Infectious Disease Specialist	44	0.0
Medical Practitioner	1406	0.9
Midwife	170	0.1
Specialist Physician	809	0.5
Social Worker	10	0.0
Non-clinical service provider	39	0.0
Not stated	99	0.1
Total	165,238	100.0

a) CSNSW Psychologists

b) Includes Enrolled nurse, Nurse practitioner, Registered Nurse

c) Includes Diagnostic Radiography, Dietitian, Diversional Therapist, Occupational Therapist, Optometrist, Physiotherapist,Podiatrist, Speech Pathologist, and other Allied Health specialist

Appointments for people who reported SHSB

Table 5 shows the number of active (i.e., alert started within a previous RSA episode and still active) or new self-harm/suicide alerts and the number of RIT alerts started during the RSA episodes. Of the 42,161 RSAs included in the study, there were 3255 (7.7%) RSAs with at least one active or new self-harm/suicide alert, and 4775 (11.3%) RSAs where at least one new RIT alert was started. Of the episodes with active or new self-harm/suicide alerts, 55.1% were episodes in which people reported SHSB. 41% of the self-harm/suicide alerts and 42.1% of RIT alerts were for people who did not report SHSB at reception. Of the active or new self-harm/suicide alerts (*n* = 1794) and new RIT alerts (*n* = 2513) from RSAs where people reported SHSB, at least one quarter (25.5% and 35.2%, respectively) were for episodes where attempts within one month prior to reception were reported (Table 5).

**Table 5:** PAS self-harm/suicide and RIT alerts

Reported SHSB at reception	At least one active or new self-harm/suicide alert		At least one new RIT alert		Both	
	*N*	%	*N*	%	*N*	%
Yes	1794	55.1	2513	52.6	694	63.3
* Attempts within one month prior to prison reception*	*458*	*25.5*	*884*	*35.2*	*265*	*38.2*
No	1333	41.0	2008	42.1	345	31.5
Not stated	128	3.9	254	5.3	57	5.2
All	3255	100.0	4775	100	1096	100.0

based on number of RSAs

*PAS* Patient Administration System, *RIT* Risk Integration Team, *SHSB* Self-harm and/or suicidal behaviour

During the study period, there were 5446 new RIT and 30,607 RIT review appointments. The number of new RIT or RIT review appointments within four weeks after reception are presented in.

Table 6. In total, there were 21,753 new RIT and RIT review appointments where the date of appointments is at or within four weeks of the reception date. Of these, 59.0% (*n* = 12,839) were following RSAs where people reported SHSB. Over a third (33.8%) were following RSAs where people did not report SHSB (Table 6). For episodes with at least one new RIT appointment, the median days from reception to the first new RIT was three days (IQR = 1) for RSAs where people reported SHSB and five (IQR = 5) days for RSAs where people did not report SHSB (*P* < 0.001). The median number of RIT review appointments within four weeks of reception was two appointments for both RSAs where people reported SHSB and those where people did not report SHSB.

**Table 6:** RIT appointments within 4 weeks after reception^a^

Reported SHSB at reception	New RIT appointments		RIT review appointments		All RIT appointments	
	*N*	%	*N*	%	*N*	%
Yes	1946	54.3	10,893	60.0	12,839	59.0
* Attempts within one month prior to prison reception*	*751*	*38.6*	*4682*	*43.0*	*5433*	*42.3*
No	1423	39.7	5924	32.6	7347	33.8
Not stated	214	6.0	1353	7.4	1567	7.2
All	3583	100.0	18,170	100.0	21,753	100.0

a) Excludes appointments for RIT termination

*RIT* Risk Integration Team, *SHSB* Self-harm and/or suicidal behaviour

### Unplanned transfer to an external hospital

Unplanned transfers to an external hospital are presented in Table 7. For the period from October 2020 to September 2024, there were 3247 unplanned transfers to an external hospital. Of these, 452 (13.9%) were patients transferred following incidents of self-harm or suspected suicide attempts. A quarter (24.8%) of the transfers for self-harm or suspected suicide attempts resulted in the patient being admitted. Of these admissions, 58.0% were admitted for less than 24 h and 30.4% were admitted for one day. Of the transfers for incidents of self-harm or suspected suicide attempts, 67.3% were people who had reported SHSB at reception and 28.1% were during episodes where people did not report a history SHSB (*p* < 0.001) (Table 7).

**Table 7:** Unplanned transfer to an external hospital

All unplanned hospital transfers	*N* = 3247	%
Unplanned hospital transfers for self-harm or suspected suicide attempt	452	13.9
Admitted	112	24.8
Non-admitted	300	66.4
Not stated	40	8.8
Length of stay^a^		
Median stay	< 24 h	
< 24 h	65	58.0
1 day	34	30.4
2–4 days	7	6.3
5 or more days	6	5.4
Reported SHSB at reception		
Yes	304	67.3
No	127	28.1
Not stated	21	4.7

a) Admitted only

*SHSB* Self-harm and/or suicidal behaviour

Data available from October 2020

There were 91 (20.1%) unplanned hospital transfers due to self-harm or suspected suicide attempts that occurred within four weeks of receptions. Of these transfers, 65 (71.4%) occurred among people who reported SHSB at reception and almost a quarter (23.1%, *n* = 21) involved people who did not report SHSB at reception. This difference in the proportions of the unplanned hospital transfers within four weeks of receptions was not statically different (*p* = 0.251).

Of the 65 unplanned hospital transfers for people who reported past SHSB at reception 38 were for people who had self-harmed or attempted suicide four weeks prior to prison entry, representing 41.8% of all hospital transfers within four weeks of reception and 58.5% of those transferred to hospital who had reported past self-harm/suicidality and had their last attempt four weeks prior to prison entry.

There were 114 unplanned hospital transfers due to self-harm or suicide attempts occurring at or within seven days of a new RIT or RIT review appointments. Of these, 73 (64%) transfers occurred on the same day of the RIT appointment, with 78.1% of these transfers occurring during an RSA episode where people reported SHSB.

## Discussion

### Key results

Results from our study show that people reported SHSB in 24.3% of the RSAs, with over one third (37.7%) of Aboriginal people entering custody reporting SHSB on at least one reception occasion. At reception, depression and anxiety were the most reported mental health conditions among people who disclosed a history of self-harm or suicidal behaviours. Of RSAs where people reported past SHSB, 30.0% were placed on RIT, suggesting the risk of self-harm or suicidal behaviour wasn’t regarded as current. People who reported SHSB were 37% more likely to have a mental health appointment booked within four weeks of their reception date, compared to people who did not report self-harm or suicidal behaviour, which is consistent with increased rates of self-harm/suicide among those with unmet mental health needs. Of all appointments (i.e., all appointment types) booked within four weeks of reception for people who reported self-harm or suicidal behaviour, only 1.5% and 0.3% were with psychiatrists and an Aboriginal Health Worker, respectively.

There were 21,753 new RIT and RIT review appointments where the appointment date was on or within four weeks of the reception date. Of these, 59.0% were following RSAs where people reported SHSB. There were 452 unplanned transfers to an external hospital due to self-harm or suspected suicide attempts. Of these hospital transfers, 91 (20.1%) occurred within four weeks of reception. A high proportion of these transferees (71.4%) had reported past self-harm and/or suicide attempts; among whom 38 (58.5%) reported self-harm or suicide attempts four weeks prior to prison entry. As in previous research, these findings highlight the first four weeks of incarceration as a period of increased risk, especially among those who have reported past self-harm or suicidality [5, 39].

### Reported history of self-harm and/or suicidal behaviour

The proportion of our study population that reported a history of SHSB (37.7%) is comparable with the proportion reporting past suicide and/or self-harm attempts (38.7%) in a study conducted in NSW prisons by Browne et al. [40]. However, the rate of reported self-harm among our study population was higher than the rate among First Nations prison entrants reported in the Australian Institute of Health and Welfare (AIHW) 2022 report on the health of people in Australia’s prisons (22.1% vs. 15%) [15]. The self-harm and suicide attempts among our study population were higher than those published by Marr et al. (2025) (22.1% vs. 19.8% and 26.9% vs. 16.0%) [41]. Our study result is also higher than the history of self-harm published by Borrschmann et al. in a study conducted in Queensland prisons [42]. Self-report of SHSB on its own has been found to be of low sensitivity in indicating prior and predicting future SHSB [40, 42]. Borrschmann et al. conducted a sensitivity analysis to assess the consistency between people in prison’s self-reported history of self-harm and their retrospective health records and found that only 38% of participants with at least one medically verified self-harm event disclosed a history of self-harm [42]. Our study results show that, among our population, 2152 people have reported ever attempting self-harm and/or suicide in one RSA and did not disclose this information in a later RSA. This result adds further evidence that reporting for SHSB at prison reception is not reliable.

People in prison may be reluctant to report a history of SHSB due to several factors, which may include fear of the stigma associated with reporting self-harm or suicidality, especially among an Aboriginal population, which also contributes to hesitancy to seek health care [42–46]. Additionally, people at reception are highly distressed and can be intoxicated or in withdrawal which can affect their ability to report histories of SHSB.

Another factor is the fear of being placed under RIT management, which can result in conditions analogous to segregation, special cell placement, intense monitoring, and the perception that disclosing self-harm may negatively affect their incarceration or delay their release [40, 42]. This particularly affects people who have previously been in prison and know the effect of reporting SHSB.

Our results show that people who report a history of SHSB are more likely to have mental health appointments scheduled, indicating that those with unreported SHSB are less likely to receive timely mental health evaluation and treatment—ultimately contributing to unmet mental health needs within this population. However, it is important to note that 71% those who were transferred to hospital following self-harm or suspected suicide attempts within four weeks of incarceration, had self-reported previously engaging in these behaviours at reception. Therefore, where Aboriginal people do self-report these behaviours at reception, safeguards ought to be more readily arranged within the first month of imprisonment.

In NSW prisons, self-reported SHSB is currently the primary means of identifying individuals at risk of self-harm or suicide. This reliance on self-reporting is largely due to two key system limitations: health records for people in custody are not linked to their community hospital or emergency department records, and each Australian state and territory operates its own prison system without health data sharing between jurisdictions [47]. These disconnected systems prevent access to potentially critical health information that could support more accurate risk assessments.

In March 2026, NSW Health will begin implementing the Single Digital Patient Record (SDPR) system. By the end of 2028, the SDPR is expected to integrate electronic patient records across all local health districts and specialty networks in the state [48]. This will enable Justice Health NSW staff to access community health records, potentially improving the identification of individuals who have presented to hospitals or emergency departments for self-harm or suspected suicide attempts.

### Mental health morbidities

Aboriginal people in contact with the justice system often have intricate health and social care needs. They are affected by multiple traumatic life events such as social exclusion, discrimination, death of family or friends – including by suicide - and physical and sexual assaults [22, 49]. Added to such trauma are stressors related to the prison environment such as solitary confinement, victimisation and lack of social support [13, 16]. These stressors are associated with psychological distress, mental health issues, loneliness and suicidality [49–51]. Among the study population who entered prison between January 2015 to January 2021 and reported a history of SHSB, over three quarters (78.1%) had prior treatment for a mental health condition. Of those, more than half (55.4%) reported a history of depression. Of those who entered prisons between February 2021 and June 2024, 76.2% reported having had depression and 77.7% of those had experienced depressive symptoms within the month prior to prison entry. The second most prevalent mental health condition among those studied who reported a history of SHSB was anxiety disorder, followed by schizophrenia and bipolar affective disorder. It is important to note; however, that people can report more than one mental health condition in one RSA. Of the RSAs of people who entered prisons between February 2021 and June 2024 and reported SHSB, 79.1% reported multiple mental health conditions. The rate of mental health (co)morbidity among those who reported SHSB is indicative of a compounding risk of suicide. A study conducted by Horváthné Pato et al. (2024) revealed that among people in prison, depression accounts for 31% of the variance in suicidal ideation [11]. Additionally, Horváthné Pato et al. showed that the combination of depression and a history of SHSB was associated with an even greater increase in this risk, indicating a need for urgent tailored intervention [11]. Mental health disorders characterised by agitation and anxiety, and poor impulse-control, have been associated with a progression from suicidal ideation to suicide attempts [9, 52], perhaps demonstrating that risk of suicide can increase when multiple factors associated with this behaviour (including co-morbid mental health conditions) are present. The combination of these risk factors has also been shown to be associated with an increased likelihood of suicide attempts after release from prison, where necessary supports and controls may not be available [11]. Rates of suicide on release from prison have been found to be even higher than in-custody, with the first four to 12 weeks post release being an especially vulnerable time [53].

### Care for people with a history of self-harm and/or suicidal behaviour in NSW public prisons

Risk assessment and management planning for people at risk of self-harm/suicide in NSW public prisons is based on a complete clinical picture including history, risk and protective factors. At Justice Health NSW when an Aboriginal person is assessed as at risk of self-harm/suicide, they are referred to safety planning supports alongside Justice Health NSW Aboriginal Service provider, wherever possible. In addition, all non-Aboriginal Justice Health NSW staff are responsible for maintaining Aboriginal cultural awareness by completing the organisation’s mandatory training.

Management of people who are identified as at risk of suicide or self-harm relies on a collaboration between Justice Health NSW and CSNSW [54]. If a person is identified as at risk of self-harm or suicide, they may be referred to the review of a RIT. The RIT consists of two CSNSW members (Custodial Officer- RIT Coordinator and Offender Services and Programs staff) and one Justice Health NSW staff (Registered Nurse – Primary Health or Mental Health Nurse). When a RIT convenes, it is responsible for ongoing review of risk and risk management by way of a reductive approach, by limiting access to means of suicide and/or self-harm. While subject to review by a RIT, individuals may also be referred for clinical assessment or treatment as needed. RIT may also refer individuals to support services when discharged from the safety conditions imposed by the RIT [54]. For the study population, more than half (59%) of new RIT and RIT review appointments were booked within four weeks of the reception date for people who self-reported a history of SHSB. For those people the median days to the first new RIT appointment was three days.

Although RIT management aims to minimise suicide risk by restricting people’s access to means of suicide, previous literature shows that conditions of isolation, which may be experienced similarly to solitary confinement, increases risk of suicide [39, 55]. Our study found that 25% of unplanned hospital transfers for self-harm or suspected suicide attempts occurred at or within 7 days of new RIT or RIT review appointments. This suggests that an at-risk population has been identified for RIT review and placement, however, the reductive approach of RIT may not be sufficiently therapeutic because a quarter of people are engaging in this behaviour despite the intention of RIT placement is to manage the risk of self-harm and suicide. Additionally, RIT is not a health intervention and is not intended to address people’s underlying risk factors for self-harm/suicide. Justice Health NSW is currently piloting a new model of care where people at risk of self-harm/suicide have access to health-led risk assessment and planning through the Suicide Prevention Outreach Team (SPOT). SPOT is a multidisciplinary service that supports people who are at-risk of suicide. This service is implemented in Justice Health NSW as part of the NSW Health Towards Zero Suicides program [56, 57].

### Risk reduction

The prison setting offers a unique environment to provide a holistic health care to people who have complex health needs and may not have the opportunity to receive health care in the community [58]. However, non-urgent health care in prison is secondary to security and control [59]. People can only be seen for a short period of time during the day, and the movement of people between correctional centres, and in and out of prison, affects health care planning and intervention [59, 60]. Moreover, the traumatic nature of the correctional system adds significant barriers to the provision of culturally safe health care. The prison environment, characterised by surveillance, restriction, and loss of autonomy can not only induce trauma but also exacerbate existing trauma, which is highly prevalent among incarcerated populations [60].

Previously published literature on suicide risk reduction among Aboriginal people highlights the importance of trauma-informed, Aboriginal-led interventions and programs, and care provided by Aboriginal health workers in reducing the risk of suicide and improving the mental health of Aboriginal people in prison and the community [35, 61–64]. Trauma-informed care acknowledges that individuals’ maladaptive behaviours are expressions of distress and trauma, making it crucial to avoid causing further trauma or reactivating past traumatic experiences [64]. Additionally, engaging Aboriginal people in programs and healthcare ensures that interventions are designed and delivered by those who understand the needs and strengths of the Aboriginal community [64]. Our results show only 0.3% of all appointments within four weeks of reception for people who reported a history of self-harm/suicidal behaviour were for an Aboriginal Health Worker. As of April 2024, Justice Health NSW, has only 7 Aboriginal health workers/clinicians representing 0.3% of Justice Health NSW staff in stark contrast to the 32.3% of Aboriginal people among NSW prison population. The low proportion of appointments for an Aboriginal Health Worker highlights a critical gap in culturally appropriate care. Aboriginal Health Workers play a vital role in delivering holistic, trauma-informed support that aligns with Aboriginal concepts of social and emotional wellbeing [65].

Additionally, the immediate period when people enter prison is crucial in reducing suicide risk. Previous literature shows that the immediate period of prison entry is a distressing time and associated with an increased risk of suicide [39, 66, 67]. The current study results show that one in five unplanned hospital transfers due to self-harm or suspected suicide attempts occurred at or within four weeks of prison reception. This highlights the importance of suicide risk assessments being booked within four weeks of reception for people who report SHSB.

The Living Is For Everyone (LIFE) Framework highlights the significance of early intervention when people are distressed and show signs and symptoms of suicidal risk [68]. *The health of people in Australia’s prisons 2022* report by AIHW shows that one third of Aboriginal prison entrants were at high or very high levels of distress at reception [69].

However, it is important to note that while the immediate period after prison entry is crucial for identifying and intervening to prevent self-harm/suicide, ongoing risk assessment throughout the incarceration period, especially around the time of significant court dates including sentencing, are similarly indicated [12, 39].

### Strength and limitations

This is the first study that explores health care provided in NSW public prisons for Aboriginal people who reported SHSB at reception. While previous studies conducted in Australian prisons investigated self-report of SHSB at reception and subsequent incidents of SHSB, our study results added information on utilisation of services to reduce the risk of incidents of SHSB such as mental health appointments and admission to RIT. In this study we utilised real-world data extracted from Justice Health NSW electronic data systems. The use of PAS data provides information based on health care activities recorded by health professionals which provide more accurate information than self-reported data used in other studies.

One limitation of this study is that we are unable to determine the timing of an individual’s release from prison from the health data alone, which may underestimate the number of events such as mental health appointments; however, focusing on the first four weeks following reception may reduce this bias. Another limitation is the lack of the unplanned transfer to an external hospital data prior to October 2020, which is likely to underestimate the number of hospital transfers that occurred for our population during the study period.

## Conclusion and future directions

Self-reporting a history of self-harm/suicide at prison reception has only modest reliability for indicating subsequent suicide/self-harm risk whilst in prison, as highlighted by the 23.1% of unplanned hospital transfers following a self-harm episode where the patient did not have a reported history of self-harm/suicide. A higher proportion of people who disclosed a history of self-harm or suicide attempts had mental health appointments organised for them, were referred to a mental health nurse and/or placed on RIT compared to those who did not, underscoring the importance of suicide/self-harm risk assessment both at reception and while a person is in custody. Better identification of people with self-harm/suicide behaviour, early intervention, creating a safe environment and providing multifaceted and culturally informed health care may contribute to reduce the risk of self-harm and suicide among people in prison.

Whilst our study is based on a quantitative data analysis which allowed us to investigate patterns service utilisation of people with a history of self-harm and suicide among Aboriginal people in custody, further research is needed to reflect the deeper impacts of colonisation, cultural disconnection, and trauma. A more holistic, Aboriginal-led approach—grounded in community engagement and centred on cultural healing and lived experience—is essential for meaningful understanding of the health care needs of Aboriginal people in prison.

## Data Availability

Datasets utilised for the analysis of this study is not for sharing as ethical approvals for this project require that the data used in this analysis not be shared to protect privacy and confidentiality.

## Abbreviations

CSNSW: Corrective Services NSW
JHeHS: Justice health electronic health system
Justice Health NSW: Justice Health and Forensic Mental Health Network
NSW: New South Wales
PAS: Patient Administration System
RIT: Risk Integration Team
RSA: Reception Screening Assessment
SHSB: Self-harm and/or suicidal behaviour

## References

[1] World Health Organisation (WHO), Suicide. WHO; 2024. https://www.who.int/news-room/fact-sheets/detail/suicide#:~:text=More%20than%20720%20000%20people,%2D%20and%20middle%2Dincome%20countries. Accessed March 11 2025.

[2] Australian Institute of Health and Welfare (AIHW). Suicide & self-harm monitoring: AIHW. 2024. https://www.aihw.gov.au/suicide-self-harm-monitoring/data/deaths-by-suicide-in-australia/suicide-deaths-over-time. Accessed March 11 2025.

[3] Australian Bureau of Statistics (ABS). Intentional self-harm deaths (Suicide) in Australia Canberra: ABS. 2024. https://www.abs.gov.au/statistics/health/causes-death/causes-death-australia/latest-release#intentional-self-harm-deaths-suicide-in-australia. Accessed May 5 2025.

[4] Australian Bureau of Statistics (ABS). Intentional self-harm deaths (Suicide) of Aboriginal and Torres Strait Islander people Canberra: ABS. 2025. https://www.abs.gov.au/statistics/health/causes-death/causes-death-australia/latest-release#intentional-self-harm-deaths-suicide-of-aboriginal-and-torres-strait-islander-people. Accessed May 5 2025.

[5] Favril L. Epidemiology, risk factors, and prevention of suicidal thoughts and behaviour in prisons: a literature review. Psychol Belg. 2021. 10.5334/pb.1072. 34900324 10.5334/pb.1072 PMC8622377

[6] World Health Organisation (WHO). Preventing suicide in jails and prisons. Geneva, Switzerland: Department of Mental Health and Substance Abuse World Health Organization; 2007.

[7] Kariminia A, Butler TG, Corben SP, Levy MH, Grant L, Kaldor JM, et al. Extreme cause-specific mortality in a cohort of adult prisoners—1988 to 2002: a data-linkage study. Int J Epidemiol. 2007;36(2):310–6. 10.1093/ije/dyl225. 17158524 10.1093/ije/dyl225

[8] Willis M, Baker A, Cussen T, Patterson E. Self-inflicted deaths in Australian prisons. Trends Issues Crime Crim Justice. 2016;513. 10.52922/ti154878.

[9] Favril L, O’Connor RC. Distinguishing prisoners who think about suicide from those who attempt suicide. Psychol Med. 2021;51(2):228–35. 10.1017/s0033291719003118. 31736457 10.1017/S0033291719003118

[10] Favril L, Indig D, Gear C, Wilhelm K. Mental disorders and risk of suicide attempt in prisoners. Soc Psychiatry Psychiatr Epidemiol. 2020;55(9):1145–55. 10.1007/s00127-020-01851-7. 32144468 10.1007/s00127-020-01851-7

[11] Horváthné Pató I, Kresznerits S, Szekeres T, Zinner-Gérecz Á, Perczel-Forintos D. Investigating suicidal behavior among prisoners in the light of the behavioral addiction approach: results of a multicenter cross-sectional study. Front Psychiatry. 2024. 10.3389/fpsyt.2024.1448711. 39119071 10.3389/fpsyt.2024.1448711 PMC11306188

[12] Zhong S, Senior M, Yu R, Perry A, Hawton K, Shaw J, et al. Risk factors for suicide in prisons: a systematic review and meta-analysis. Lancet Public Health. 2021;6(3):e164–74. 10.1016/S2468-2667(20)30233-4. 33577780 10.1016/S2468-2667(20)30233-4 PMC7907684

[13] Favril L, Shaw J, Fazel S. Prevalence and risk factors for suicide attempts in prison. Clin Psychol Rev. 2022;97:102190. 10.1016/j.cpr.2022.102190. 36029609 10.1016/j.cpr.2022.102190

[14] Justice Health and Forensic Mental Health Network. Network Patient Health Survey. 2017.

[15] Australian Institute of Health and Welfare. The health of people in australia’s prisons 2022. Canberra: Australian Government; 2023. Report No.: catalogue number PHE 334.

[16] Favril L, Yu R, Hawton K, Fazel S. Risk factors for self-harm in prison: a systematic review and meta-analysis. Lancet Psychiatry. 2020;7(8):682–91. 10.1016/s2215-0366(20)30190-5. 32711709 10.1016/S2215-0366(20)30190-5 PMC7606912

[17] Poorolajal J, Haghtalab T, Farhadi M, Darvishi N. Substance use disorder and risk of suicidal ideation, suicide attempt and suicide death: a meta-analysis. J Public Health (Oxf). 2016;38(3):e282–91. 10.1093/pubmed/fdv148. 26503486 10.1093/pubmed/fdv148

[18] Daniel AE. Preventing suicide in prison: a collaborative responsibility of administrative, custodial, and clinical staff. J Am Acad Psychiatry Law. 2006;34(2):165. 16844795

[19] Australian Bureau of Statistics (ABS). Prisoners in Australia Canberra: ABS. 2024. https://www.abs.gov.au/statistics/people/crime-and-justice/prisoners-australia/latest-release#aboriginal-and-torres-strait-islander-prisoners. Accessed March 11 2025.

[20] Australian Bureau of Statistics (ABS). Estimates of Aboriginal and Torres Strait Islander Australians Canberra. Australia ABS. 2023. https://www.abs.gov.au/statistics/people/aboriginal-and-torres-strait-islander-peoples/estimates-aboriginal-and-torres-strait-islander-australians/latest-release. Accessed Feb 15 2025.

[21] Dudgeon P, Calma T, Holland C. The context and causes of the suicide of Indigenous people in Australia. J Indig Wellbeing. 2017;2(2):5–15.

[22] Pandeya NA, Schluter PJ, Spurling GK, Tyson C, Hayman NE, Askew DA. Factors associated with thoughts of Self-Harm or suicide among aboriginal and Torres Strait Islander people presenting to urban primary care: an analysis of De-Identified clinical data. Int J Environ Res Public Health. 2022;19(1). 10.3390/ijerph19010153. 10.3390/ijerph19010153 PMC8750353 35010413

[23] Kirmayer LJ, Brass GM, Holton T, Paul K, Simpson C, Tait CL. Suicide Among Aboriginal People in Canada. Ottawa: Aboriginal Healing Foundation; 2007.

[24] Australian Institute of Criminology. Deaths in Custody in Australia 2022–23. Canberra: Institute of Criminology; 2023.

[25] NSW Office of the State Coroner. REPORT BY THE NSW STATE CORONER INTO First. Nations people’s deaths in custody in NSW. New South Wales, Australia: The NSW Coroners Court; 2021.

[26] Corrective Services NSW. Recommendation 178 – Training and development for staff [Internet] Sydney: NSW Department of Communities and Justice. https://dcj.nsw.gov.au/content/dcj/csnsw/csnsw-home/resources/research-and-reports/deaths-in-custody/review-recommendations-rciadic/training-and-development-for-staff/recommendation-178.html. Accessed Sep 8 2025.

[27] Crimes. (Administration of Sentences) Regulation 2014 (NSW), (2014).

[28] Productivity Commssion. Report on government services 2025: corrective services. Canberra; 2025.

[29] Corrective Services NSW. Visits to inmates by family and friends (Custodial operations policy and procedures 10.1). Corrective Services NSW; 2017.

[30] Barkworth J, Heinecke K, Thaler O, Howard M. Implementing digital technologies in prison: inmates’ ongoing experiences of tablet access and connections with family and friends. Corrective Services NSW; 2024. Report No.: Research Brief No. 20.

[31] Corrective Services NSW. In: Justice, DoCa, editors. Compendium of offender behaviour change programs. New South Wales, Australia: Department of Communities and Justice; 2023.

[32] Justice Health and Forensic Mental Health Network. 10 Year Strategic Plan 2023-32. Justice Health and Forensic Mental Health Network; 2022.

[33] Biles D, McDonald D, Fleming J. Aboriginal and non-aboriginal deaths in custody. Aust N Z J Criminol. 1990;23(1):15–23. 10.1177/000486589002300102.

[34] Black EB, Kisely S. A systematic review: non-suicidal self-injury in Australia and New Zealand’s Indigenous populations. Aust Psychol. 2018;53(1):3–12. 10.1111/ap.12274.

[35] Nathan S, Maru K, Williams M, Palmer K, Rawstorne P. Koori voices: self-harm, suicide attempts, arrests and substance use among Aboriginal and Torres Strait Islander adolescents following residential treatment. Health Justice. 2020;8(1):4. 10.1186/s40352-020-0105-x. 32034568 10.1186/s40352-020-0105-x PMC7007640

[36] Rasmussen MK, Donoghue DA, Sheehan NW. Suicide /self-harm-risk reducing effects of an aboriginal Art program for aboriginal prisoners. Adv Ment Health. 2018;16(2):141–51. 10.1080/18387357.2017.1413950.

[37] Reser JP. Australian aboriginal suicide deaths in custody: cultural context and cluster evidence. Aust Psychol. 1989;24(3):325–42. 10.1080/00050068908259573.

[38] Hosmer DW, Lemeshow S, Rodney X, Sturdivant. Applied logistic regression. Third ed: Wiley; 2013.

[39] Favril L, Wittouck C, Audenaert K, Vander Laenen F. A 17-year national study of prison suicides in Belgium. Crisis. 2018;40(1):42–53. 10.1027/0227-5910/a000531. 30052079 10.1027/0227-5910/a000531

[40] Browne C, Chemjong P, Korobanova D, Jang S, Yee N, Marr C, et al. Self-harm risk screening on prison entry: assessing the predictive validity of self-harm history and recent ideation in men and women. Int J Prison Health. 2023;19(3):414–26. 10.1108/ijph-12-2021-0115. 36422644 10.1108/IJPH-12-2021-0115

[41] Marr C, Browne C, Ngui D, Zeki R, Woods E, Dean K. Mental health and self-harm/suicide risk screening at prison entry over 12 months in a total population sample in New South Wales, Australia. Aust N Z J Psychiatry. 2025;59(7):629–39. 10.1177/00048674251336031. 40292451 10.1177/00048674251336031 PMC12181648

[42] Borschmann R, Young JT, Moran P, Spittal MJ, Snow K, Mok K, et al. Accuracy and predictive value of incarcerated adults’ accounts of their self-harm histories: findings froman Australian prospective data linkage study. CMAJ Open. 2017;5(3):E694-e701. 10.9778/cmajo.20170058. 28893844 10.9778/cmajo.20170058 PMC5621944

[43] Martin G, Swannell S, Harrison J, Hazell P, Taylor A. The Australian National epidemiological study of Self-Injury (ANESSI). Brisbane, Australia: Centre for Suicide Prevention Studies; 2010.

[44] Farrelly T, Francis K. Definitions of suicide and self-harm behavior in an Australian aboriginal community. Suicide Life Threat Behav. 2009;39(2):182–9. 10.1521/suli.2009.39.2.182. 19527158 10.1521/suli.2009.39.2.182

[45] Wyllie JM, Robb KA, Sandford D, Etherson ME, Belkadi N, O’Connor RC. Suicide-related stigma and its relationship with help-seeking, mental health, suicidality and grief: scoping review. BJPsych Open. 2025;11(2):e60. 10.1192/bjo.2024.857. 40116563 10.1192/bjo.2024.857 PMC12001961

[46] Heard TR, McGill K, Skehan J, Rose B. The ripple effect, silence and powerlessness: hidden barriers to discussing suicide in Australian aboriginal communities. BMC Psychol. 2022;10(1):23. 10.1186/s40359-022-00724-9. 35130962 10.1186/s40359-022-00724-9 PMC8822784

[47] Scheibner J, Kroesche N, Wakefield L, Cockburn T, McPhail SM, Richards B. Does legislation impede data sharing in Australia across institutions and jurisdictions? A scoping review. J Med Syst. 2023;47(1):116. 10.1007/s10916-023-02009-z. 37962613 10.1007/s10916-023-02009-z

[48] NSW Health. Single Digital Patient Record (SDPR): NSW Government. 2025. https://www.health.nsw.gov.au/single-digital-patient-record/Pages/default.aspx. Accessed September 11 2025.

[49] Shepherd SM, Spivak B, Arabena K, Paradies Y. Identifying the prevalence and predictors of suicidal behaviours for Indigenous males in custody. BMC Public Health. 2018;18(1):1159. 10.1186/s12889-018-6074-5. 30286743 10.1186/s12889-018-6074-5 PMC6172717

[50] Moore KE, Siebert S, Brown G, Felton J, Johnson JE. Stressful life events among incarcerated women and men: association with depression, loneliness, hopelessness, and suicidality. Health Justice. 2021;9(1):22. 10.1186/s40352-021-00140-y. 34427798 10.1186/s40352-021-00140-y PMC8386053

[51] Carlson BE, Shafer MS. Traumatic histories and stressful life events of incarcerated parents: childhood and adult trauma histories. Prison J. 2010;90(4):475–93. 10.1177/0032885510382224.

[52] Nock MK, Hwang I, Sampson NA, Kessler RC. Mental disorders, comorbidity and suicidal behavior: results from the National Comorbidity Survey Replication. Mol Psychiatry. 2010;15(8):868–76. 10.1038/mp.2009.29. 19337207 10.1038/mp.2009.29 PMC2889009

[53] Miller TR, Weinstock LM, Ahmedani BK, Carlson NN, Sperber K, Cook BL, et al. Share of adult suicides after recent jail release. JAMA Netw Open. 2024;7(5):e249965–e. 10.1001/jamanetworkopen.2024.9965. 38728036 10.1001/jamanetworkopen.2024.9965 PMC11087834

[54] Corrective Services NSW. Management of inmates at risk of self-harm or suicide (Custodial operations policy and procedures 3.7). Corrective Services NSW; 2017.

[55] Fazel S, Cartwright J, Norman-Nott A, Hawton K. Suicide in prisoners: a systematic review of risk factors. J Clin Psychiatry. 2008;69(11):1721–31. 19026254

[56] NSW Health. Towards Zero Suicides: NSW government https://www.health.nsw.gov.au/towardszerosuicides. Accessed March 17 2025.

[57] NSW Health. Suicide Prevention Outreach Teams: NSW Government. https://www.health.nsw.gov.au/towardszerosuicides/Pages/suicide-prevention-outreach-teams.aspx#spot. Accessed March 17 2025.

[58] Fazel S, Baillargeon J. The health of prisoners. Lancet. 2011;377(9769):956–65. 10.1016/s0140-6736(10)61053-7. 21093904 10.1016/S0140-6736(10)61053-7

[59] Hampton S, Abbott P. Out of sight, out of mind: investing in prison primary healthcare to target vulnerable groups. Aust J Gen Pract. 2025;54:234–5. 40174625 10.31128/AJGP-09-24-7397

[60] Nous Group. The National Review of First Nations Health Care in Prisons: Final Report Canberra; 2024.

[61] Clifford AC, Doran CM, Tsey K. A systematic review of suicide prevention interventions targeting Indigenous peoples in Australia, united States, Canada and New Zealand. BMC Public Health. 2013;13(1):463. 10.1186/1471-2458-13-463. 23663493 10.1186/1471-2458-13-463 PMC3663804

[62] Australian Institute of Health and Welfare (AIHW). Improving mental health outcomes for Indigenous Australians in the criminal justice system. Cat. no. IMH 2. Canberra: AIHW; 2021.

[63] Truong M, Moore E. Racism and Indigenous wellbeing, mental health and suicide. Catalogue number IMH 17. Australian Government; 2023.

[64] Wright M, Davison S, Petch E. Making visible the invisible: Aboriginal forensic mental health. Lancet Psychiatry. 2017;4(12):895–6. 10.1016/S2215-0366(17)30234-1. 28943186 10.1016/S2215-0366(17)30234-1

[65] NSW Ministry of Health. NSW aboriginal mental health and wellbeing strategy 2020–2025. New South Wales, Australia: NSW Ministry of Health; 2020.

[66] Radeloff D, Ten Hövel M, Brennecke G, Stoeber FS, Lempp T, Kettner M, et al. Suicide after reception into prison: a case-control study examining differences in early and late events. PLoS One. 2021;16(8):e0255284. 10.1371/journal.pone.0255284. 34343175 10.1371/journal.pone.0255284 PMC8330938

[67] Marzano L, Hawton K, Rivlin A, Smith EN, Piper M, Fazel S. Prevention of suicidal behavior in prisons. Crisis. 2016;37(5):323–34. 10.1027/0227-5910/a000394. 27278569 10.1027/0227-5910/a000394 PMC5120691

[68] Department of Health and Aging. Living Is For Everyone (LIFE) Framework. Commonwealth of Australia; 2007.

[69] Australian Institute of Health and Welfare. The health of people in Australia’s prisons 2022: At glance Canberra Australian Government 2023. https://www.aihw.gov.au/getmedia/51d38622-c670-46b2-9bed-935c6fd8ee51/aihw-phe-334-the-health-of-people-in-australias-prison-2022-at-a-glance.pdf

